# A Case of Severe Advanced Diabetic Cardiac Autonomic Neuropathy: Severe Orthostatic Hypotension Complicated With Episodes of Nocturnal Supine Hypertensive Emergency Episodes

**DOI:** 10.7759/cureus.75153

**Published:** 2024-12-05

**Authors:** Nyan Myint

**Affiliations:** 1 Internal Medicine, Meharry Medical College, Nashville, USA

**Keywords:** cardiac autonomic dysfunction, diabetic cardiovascular autonomic neuropathy, hypertensive crises, postural orthostatic hypotension, recurrent syncope, secondary cardiac dysautonomia, supine hypertension-orthostatic hypotension (sh-oh)

## Abstract

Diabetic cardiac autonomic neuropathy (CAN) is caused by damage to the autonomic nerve fibers that innervate the heart and blood vessels, leading to abnormalities in heart rate control and vascular dynamics. CAN encompasses symptoms such as exercise intolerance, orthostatic hypotension, cardiac denervation syndrome, and nocturnal hypertension. Neurogenic orthostatic hypotension (nOH), resulting from severe diabetic CAN, can cause symptomatic orthostatic hypotension.

The management of orthostatic hypotension primarily focuses on preventing severe symptoms, such as syncope and falls. It is equally important to address nocturnal supine hypertension, as it can exacerbate morning orthostatic hypotension. The management strategies for these conditions often complicate each other, highlighting the intricate and delicate nature of treating severe orthostatic hypotension associated with diabetic CAN. We present the case of a 55-year-old male with symptomatic orthostatic hypotension and coexisting nocturnal supine hypertension caused by severe, advanced diabetic CAN.

## Introduction

Diabetes mellitus (DM) mainly causes premature deaths through increased risk of cardiovascular diseases. Progression of DM is also associated with chronic complications including neuropathy and cardiomyopathy. Diabetic cardiac autonomic neuropathy (CAN) can result in abnormal heart rate variations and orthostatic hypotension [1]. 

Neurogenic orthostatic hypotension (nOH) is regarded to be one of the symptoms of severe diabetic CAN. This condition's management should incorporate both nonpharmacological and pharmacological strategies to alleviate symptoms. Nocturnal supine hypertension is also a common problem in patients with severe diabetic CAN and it can present a therapeutic dilemma in the management of nOH [2]. 

## Case presentation

A 55-year-old male with a medical history of insulin-dependent type 2 DM (diagnosed 20 years ago), dyslipidemia, and coronary artery disease (status post three-vessel coronary artery bypass graft in 2004) was admitted to a tertiary care center following the administration of tenecteplase for acute left-sided weakness. The patient presented with left hemiparesis, left hemihypoesthesia, left central facial palsy, and dysarthria, all of which occurred less than 3 hours before receiving tenecteplase. At the time of administration, his National Institutes of Health Stroke Scale (NIHSS) score was 6. Upon arrival at the hospital, the patient reported subjective improvement in his motor weakness. He also noted that his blood glucose levels had been in the 50s mg/dL, as he was using continuous glucose monitoring with a home glycemic control regimen, including a basal-bolus insulin approach, for his poorly controlled diabetes. This low glucose level was unusual for him, and he speculated that it might have contributed to his presenting symptoms.

Upon arrival, vital signs were as follows: blood pressure 161/87 mmHg, heart rate 87 bpm, respiratory rate 16 breaths per minute, oxygen saturation 97% on room air, and temperature 98.1°F. Physical examination revealed an intact mental status, left central facial palsy, mild left hemiparesis (4/5 motor strength in the left arm and 4+/5 motor strength in the left leg), and mild left hemihypoesthesia (C6, C7, C8, T1 dermatomes, except for the trunk of the left arm; L3, L4, L5, S1, S2 dermatomes, except for the saddle region). The remainder of the physical examination was unremarkable.

Laboratory results revealed the following: white blood cells 8.17 x 10^9^/L, hemoglobin 12.9 g/dL, hematocrit 38.4%, platelets 232 x 10^9^/L, sodium 139 mmol/L, potassium 3.9 mmol/L, chloride 108 mmol/L, bicarbonate 22.2 mmol/L, glucose 246 mg/dL, urea 42 mg/dL, creatinine 1.61 mg/dL, estimated glomerular filtration rate 50 mL/min/1.73 m², calcium 9.5 mg/dL, total cholesterol 245 mg/dL, low-density lipoprotein (LDL) 178 mg/dL, and hemoglobin A1c 11.9%. Table 1 shows the admission laboratory results with reference ranges.

**Table 1:** Admission lab results with reference ranges eGFR: estimated glomerular filtration rate; /L: per liter; g/dL: grams per deciliter; mmol/L: millimoles per liter; mg/dL: milligrams per deciliter; mL/min/1.73m²: milliliter per minute per 1.73 meters squared; LDL: low-density lipoprotein; HbA1c: hemoglobin A1c.

Parameter	Value	Unit	Reference range
White cell count	8.17	x10^9^/L	4.0-11.0
Hemoglobin	12.9	g/dL	13.8-17.2
Hematocrit	38.4	%	40-50
Platelets	232	x10^9^/L	150-400
Sodium	139	mmol/L	135-145
Potassium	3.9	mmol/L	3.5-5.0
Chloride	108	mmol/L	98-106
Bicarbonate	22.2	mmol/L	22-29
Glucose	246	mg/dL	70-100
Urea	42	mg/dL	7-20
Creatinine	1.61	mg/dL	0.6-1.2
eGFR	50	mL/min/1.73 m²	>60
Calcium	9.5	mg/dL	8.5-10.5
Total cholesterol	245	mg/dL	<200
LDL	178	mg/dL	<100 (optimal)
HbA1c	11.9	%	<5.7

Brain magnetic resonance imaging (MRI) and computed tomography angiography (CTA) of the head and neck were performed to rule out acute ischemic stroke and vascular occlusions in the brain and carotid arteries. The brain MRI did not reveal any acute ischemic infarction, and the CTA of the head and neck demonstrated patent vessels.

The left hemiparesis, hemihypoesthesia, left central facial palsy, and dysarthria resolved upon reassessment 4 hours after arrival. Dual antiplatelet therapy (aspirin 81 mg daily and clopidogrel 75 mg daily) was initiated the following day for transient ischemic attack management, along with the patient’s home medications, rosuvastatin and ezetimibe, once concerns regarding dysphagia were resolved through a swallow evaluation. Glycemic control was optimized using a sliding scale for insulin and glargine, with a reduced home dosage due to documented low blood glucose on arrival and noncompliance with the diabetic diet and insulin regimen at home. Throughout the hospital course, no hypoglycemic symptoms or concerning low blood glucose levels were observed. Glucose levels were checked four times daily, with values ranging from 100 to 220 mg/dL.

Home medications for orthostatic hypotension, including fludrocortisone, droxidopa, and pyridostigmine, were initially withheld due to the patient's hypertensive state upon admission. However, 24 hours after admission, the patient experienced dizziness and nearly lost consciousness while ambulating with the physical therapy team. Orthostatic vital signs were as follows: supine blood pressure of 126/76 mmHg with a heart rate of 76 bpm; sitting blood pressure of 109/73 mmHg with a heart rate of 78 bpm; and standing blood pressure of 67/47 mmHg with a heart rate of 83 bpm. These measurements revealed a drop of 59 mmHg in systolic blood pressure (SBP) and 29 mmHg in diastolic blood pressure (DBP), which was consistent with orthostatic hypotension.

The patient has a history of nOH, including recurrent episodes of syncope and a diagnosis of autonomic failure for over 10 years, several years after the diagnosis of type 2 DM. Back then, he was diagnosed with CAN, as evidenced by two positive cardiac autonomic reflex tests (CARTs), which showed abnormal heart rate and blood pressure responses upon standing. Diabetes was presumed to be the cause, as the patient does not have known diagnoses or signs of other synucleinopathies, such as Parkinson's disease, multiple system atrophy, or Lewy body dementia. His condition has progressively worsened, with an average of 3-4 falls per week prior to admission, sometimes accompanied by loss of consciousness, despite being on droxidopa 600 mg three times daily, fludrocortisone 0.2 mg in the morning, and pyridostigmine 60 mg three times daily. The patient had previously tried midodrine but discontinued it due to adverse reactions. There were concerns regarding medication compliance, as the patient occasionally withheld doses of droxidopa and pyridostigmine due to frequent headaches associated with supine hypertension. Given the patient's known autonomic failure, the autonomics team was consulted for inpatient management and for the reintroduction of his home medications for orthostatic hypotension.

Management of autonomic failure must focus on addressing orthostatic hypotension, which can exacerbate nocturnal supine hypertension. The primary treatment goal is to prevent symptomatic orthostasis, thereby reducing the risk of recurrent falls. Given the questionable compliance with medications and symptomatic nocturnal supine hypertension on the other hand, restarting his home orthostatic medications alongside management of nocturnal supine hypertension was necessary. Table 2 outlines the daily orthostatic vitals and the management plan for orthostatic hypotension until discharge.

**Table 2:** Daily orthostatic vitals and daily management for the orthostatic hypotension Anti-hypertensives received by the patient on the night before for nocturnal supine hypertension/hypertensive emergency episodes were added in the comment section since these can affect orthostatic hypotension management. Summary of the table: The patient's orthostatic symptoms were controlled through a combination of strict nonpharmacological measures and four medications. Three home medications (fludrocortisone, pyridostigmine, and droxidopa) were restarted at the same doses as before admission, and one new medication, atomoxetine, was initiated. Notably, orthostatic blood pressure drops were more severe when the patient had a hypertensive emergency the night before or when guanfacine was used. mmHg: millimeters of mercury; /min: per minute; mg: milligrams; am: before noon; pm: after noon; BID: two times daily; TID: three times daily.

Days since admission	Supine blood pressure in mmHg and heart rate	Sitting blood pressure in mmHg and heart rate	Standing blood pressure in mmHg and heart rate	Management for orthostasis	Comments
Day 1	147/86, 80/min	-	-	Held home orthostatic medications: 1. Droxidopa 600 mg TID. 2. Fludrocortisone 0.2 mg daily. 3. Pyridostigmine 60 mg TID.	Stable without treatment.
Day 2	126/76, 76/min	109/73, 78/min	67/47, 83/min	Conservative measures include compression stockings except for bedtime and restarted home droxidopa 600 mg TID.	Symptomatic and autonomics were consulted.
Day 3	122/75, 77/min	103/69, 80/min	76/48, 86/min	Continue droxidopa 600 mg TID and restart reduced home fludrocortisone 0.1 mg daily.	Symptomatic.
Day 4	175/90, 80/min	132/86, 77/min	92/63, 86/min	Continue droxidopa 600 mg TID and fludrocortisone 0.1 mg daily.	Received losartan 25 mg overnight.
Day 5	142/78, 78/min	118/74, 81/min	77/51, 86/min	Continue droxidopa 600 mg TID and increase fludrocortisone to 0.2 mg daily.	Received losartan 50 mg, nitro-paste, and nifedipine XL 60 mg overnight for a hypertensive emergency.
Day 6	154/90, 72/min	106/72, 79/min	81/54, 80/min	Continue droxidopa 600 mg TID, fludrocortisone 0.2 mg daily, and restart pyridostigmine 60 mg TID.	Received losartan 50 mg overnight.
Day 7	178/98, 69/min	138/90, 74/min	96/64, 78/min	Continue droxidopa 600 mg TID, fludrocortisone 0.2 mg daily, and pyridostigmine 60 mg TID.	Received losartan 75 mg overnight.
Day 8	212/118, 66/min	169/113, 85/min	120/81, 80/min	Continue droxidopa 600 mg TID, fludrocortisone 0.2 mg daily, and pyridostigmine 60 mg TID. Start spacing out orthostatic medications.	Received losartan 75 mg, nifedipine 30 mg, and nitro-paste overnight for a hypertensive emergency.
Day 9	179/100, 67/min	119/104, 76/min	92/63, 72/min	Continue droxidopa 600 mg TID (6 am-12 pm-6 pm), fludrocortisone 0.2 mg 6 am daily, and pyridostigmine 60 mg TID (7 am-12 pm-5 pm).	Received losartan 75 mg and guanfacine 0.5 mg overnight.
Day 10	118/73, 79/min	81/57, 79/min	Cannot tolerate	Continue droxidopa 600 mg TID (6 am-12 pm-6 pm), fludrocortisone 0.2 mg 6 am daily, and pyridostigmine 60 mg TID (7 am-12 pm-5 pm). Hold guanfacine.	Received losartan 75 mg and guanfacine 0.5 mg overnight.
Day 11	192/97, 59/min	117/77, 65/min	86/57, 70/min	Continue droxidopa 600 mg TID (6 am-12 pm-6 pm), fludrocortisone 0.2 mg 6 am daily, pyridostigmine 60 mg TID (7 am-12 pm-5 pm), and start atomoxetine 10 mg BID at breakfast and lunch.	Received losartan 100 mg and nitro patch overnight.
Day 12	190/96, 67/min	153/95, 70/min	106/70, 73/min	Continue droxidopa 600 mg TID (6 am-12 pm-6 pm), fludrocortisone 0.2 mg 6 am daily, pyridostigmine 60 mg TID (7 am-12 pm-5 pm), and atomoxetine 10 mg breakfast and lunch.	Received losartan 100 mg and nitro patch overnight.
Day 13	205/103, 70/min	172/100, 74/min	128/87, 77/min	Continue droxidopa 600 mg TID (6 am-12 pm-6 pm), fludrocortisone 0.2 mg 6 am daily, pyridostigmine 60 mg TID (7 am-12 pm-5 pm), and atomoxetine 10 mg breakfast and lunch.	Received losartan 100 mg overnight.
Day 14	147/87, 72/min	90/62, 77/min	71/46, 82/min	Continue droxidopa 600 mg TID (6 am-12 pm-6 pm), fludrocortisone 0.2 mg 6 am daily, pyridostigmine 60 mg TID (7 am-12 pm-5 pm), and atomoxetine 10 mg breakfast and lunch.	Received losartan 100 mg and nitro patch overnight for a hypertensive emergency.
Day 15	175/98, 70/min	113/77, 75/min	89/58, 76/min	Continue droxidopa 600 mg TID (6 am-12 pm-6 pm), fludrocortisone 0.2 mg 6 am daily, pyridostigmine 60 mg TID (7 am-12 pm-5 pm), and atomoxetine 10 mg breakfast and lunch.	Received losartan 100 mg and nitro paste overnight for a hypertensive emergency.
Day 16	204/115, 81/min	147/90, 81/min	84/61, 88/min	Continue droxidopa 600 mg TID (6 am-12 pm-6 pm), fludrocortisone 0.2 mg 6 am daily, pyridostigmine 60 mg TID (7 am-12 pm-5 pm), and atomoxetine 10 mg breakfast and lunch.	Received losartan 100 mg and diltiazem 30 mg overnight.
Day 17	167/100, 72/min	126/84, 76/min	84/56, 80/min	Ensure the head of the bed elevation is at least 30 degrees while lying on the bed, and abdominal binder and compression stockings are removed only at bedtime. Continue droxidopa 600 mg TID (6 am-12 pm-6 pm), fludrocortisone 0.2 mg 6 am daily, pyridostigmine 60 mg TID (7 am-12 pm-5 pm), and increase atomoxetine to 18 mg BID at breakfast and lunch. Avoid pressors 3 hours before bedtime.	Received losartan 100 mg, diltiazem 30 mg, and nitro patch overnight for a hypertensive emergency.
Day 18	185/86, 89/min	149/91, 91/min	110/71, 93/min	Ensure the head of the bed elevation is at least 30 degrees while lying on the bed, and abdominal binder and compression stockings are removed only at bedtime. Continue droxidopa 600 mg TID (6 am-12 pm-6 pm), fludrocortisone 0.2 mg 6 am daily, pyridostigmine 60 mg TID (7 am-12 pm-5 pm), and atomoxetine 18 mg BID at breakfast and lunch. Avoid pressors 3 hours before bedtime.	Asymptomatic and received losartan 100 mg and short-acting nifedipine 20 mg overnight.
Day 19	172/88, 71/min	140/86, 74/min	112/68, 78/min	Ensure the head of the bed elevation is at least 30 degrees while lying on the bed, and abdominal binder and compression stockings are removed only at bedtime. Continue droxidopa 600 mg TID (6 am-12 pm-6 pm), fludrocortisone 0.2 mg 6 am daily, pyridostigmine 60 mg TID (7 am-12 pm-5 pm), and atomoxetine 18 mg BID at breakfast and lunch. Avoid pressors 3 hours before bedtime.	Asymptomatic and received losartan 100 mg and short-acting nifedipine 20 mg overnight.
Day 20	164/91, 82/min	132/82, 85/min	108/64, 86/min	Discharge and plan: 1. droxidopa 600 mg TID (6 am-12 pm-6 pm), 2. fludrocortisone 0.2 mg 6 am daily, 3. pyridostigmine 60 mg TID (7 am-12 pm-5 pm), 4. atomoxetine 18 mg BID at breakfast and lunch. Conservative measures such as ensuring the head of the bed elevation is at least 30 degrees while lying on the bed, and abdominal binder and compression stockings are removed only at bedtime. Avoid abrupt standing and pressors 3 hours from bedtime.	Asymptomatic and received losartan 100 mg and short-acting nifedipine 20 mg overnight.

The pharmacological management of orthostatic hypotension may exacerbate nocturnal supine hypertension, potentially leading to severe hypertension and symptoms that could be classified as hypertensive emergencies. To address this issue, regular antihypertensive medications were administered at bedtime. However, the patient demonstrated noncompliance with head-of-bed elevation due to reported discomfort. Notably, the patient had been experiencing frequent headaches related to nocturnal supine hypertension even prior to admission.

The treatment goal for managing nocturnal supine hypertension was to maintain SBP below 160 mmHg while ensuring that the management strategy did not worsen orthostatic hypotension. Table 3 provides a day-by-day record of nocturnal supine blood pressures and the medications administered.

**Table 3:** Day-by-day supine blood pressure checks and management Orthostatic medication changes are added in the comments as they can exacerbate nocturnal supine hypertension. Hypertensive emergency episodes symptoms, management, and work-ups will be in the next table. Summary of the table: Regular medications were planned in addition to nonpharmacological measures to control nocturnal supine hypertension. Dose escalation and the addition of another medication were implemented after each hypertensive emergency episode, except for the first one, which was attributed to the reintroduction of orthostatic medications. Losartan was escalated to the maximum dose and continued as a preferred regular bedtime antihypertensive due to the patient's underlying diabetes. A trial of guanfacine achieved nocturnal blood pressure control but was discontinued due to the precipitation of pre-syncopal falls and worsening orthostatic symptoms. Trials of nitroglycerin patch and diltiazem did not result in effective nocturnal blood pressure control. Ultimately, the patient's nocturnal supine hypertension was successfully managed with regular evening doses of losartan 100 mg and nifedipine short-acting 20 mg. mg: milligrams; am: before noon; BID: two times a day; TID: three times a day; mg/h: milligrams/hour.

Days since admission	Bedtime supine blood pressure in mmHg before medications	Early morning blood pressure in mmHg	Planned anti-hypertensives given in the evening (9 pm bedtime)	Comments
Day 1	167/99	155/86	-	-
Day 2	166/97	178/96	-	Restarted home droxidopa 600 mg TID in the am
Day 3	162/89	156/92	Losartan 25 mg	Restarted home fludrocortisone 0.1 mg in the am
Day 4	213/113	164/93	Losartan 50 mg	First-time hypertensive emergency episode
Day 5	131/72	162/90	Losartan 50 mg	Increased fludrocortisone from 0.1 mg to 0.2 mg
Day 6	213/103	162/86	Losartan 50 mg	Restarted home pyridostigmine 60 mg TID in the am and losartan 25 mg given on top of losartan 50 mg at bedtime
Day 7	238/118	166/106	Losartan 75 mg	Second-time hypertensive emergency episode
Day 8	193/103	120/69	Losartan 75 mg and guanfacine 0.5 mg BID	Guanfacine 0.5 mg BID started at bedtime and started spacing the orthostatic medications during the daytime
Day 9	191/104	117/73	Losartan 75 mg and guanfacine 0.5 mg BID	Pre-syncopal fall in early am while ambulating
Day 10	200/99	139/72	Losartan 100 mg and transdermal nitroglycerin patch 0.1 mg/h	Discontinued guanfacine 0.5 mg BID but already received am dose
Day 11	147/84	157/84	Losartan 100 mg and transdermal nitroglycerin patch 0.1 mg/h	Started atomoxetine 10 mg BID with breakfast and lunch
Day 12	161/95	174/97	Losartan 100 mg	-
Day 13	242/119	148/80	Losartan 100 mg	Third-time hypertensive emergency episode
Day 14	168/92	163/103	Losartan 100 mg and transdermal nitroglycerin patch 0.2 mg/h	Fourth-time hypertensive emergency episode at 1 am between checks with blood pressure of 236/106
Day 15	161/99	156/92	Losartan 100 mg and diltiazem 30 mg	-
Day 16	233/109	164/92	Losartan 100 mg and diltiazem 30 mg	Fifth-time hypertensive emergency episode
Day 17	155/76	164/95	Losartan 100 mg and short-acting nifedipine 20 mg	Increased atomoxetine 18 mg BID breakfast and lunch in the am
Day 18	198/93	159/81	Losartan 100 mg and short-acting nifedipine 20 mg	-
Day 19	182/94	122/72	Losartan 100 mg and short-acting nifedipine 20 mg	-

Table 4 shows the hypertensive emergency episodes and the management provided to resolve those episodes.

**Table 4:** Hypertensive emergency episodes while inpatient and management Since the symptoms during these episodes were similar to the initial presentation and the patient had never been diagnosed with nocturnal supine hypertension, these episodes were attributed to a worsening of his cardiac autonomic neuropathy, in addition to the side effects of his orthostatic medications. Other potential causes of secondary hypertension, such as pheochromocytoma and hyperaldosteronism, were considered. However, the symptoms and uncontrolled blood pressures were restricted to the supine position (bedtime), and normal potassium and bicarbonate levels reduced the suspicion of these conditions. XL: extra-long acting; EKG: electrocardiogram; CT: computed tomography; mg/h: milligrams per hour; am: before noon.

Hypertensive episodes	Blood pressure and symptoms	Management	Work-ups, recheck blood pressure, and patient’s conditions
First time (day 4 since admission)	Awoken around 11 pm with a blood pressure of 213/113 with headache, left-sided upper extremity weakness and tingling, and chest pain.	Already took losartan 25 mg in the evening. Nitro-paste and nifedipine XL 60 mg were given. Elevate the head of the bed to 30 degrees. Troponin and EKG were ordered. CT head w/o contrast was ordered.	Recheck blood pressure after 1 hour was 168/112. Recheck blood pressure after 2 hours was 164/93. The symptoms resolved after 1 hour. Troponins were negative, EKG did not show any ST segment, T, and Q wave changes, and CT head showed no acute intracranial bleed.
Second time (day 7 since admission)	Bedtime blood pressure check was 238/118 with headache, left-sided upper extremity weakness with tingling, nausea, vomiting, and chest pain.	Already took losartan 50 mg in the evening. Nitro-paste and nifedipine 30 mg were given. Elevate the head of the bed to 30 degrees. Troponin and EKG were ordered.	Recheck blood pressure after 1 hour was 178/110. Recheck blood pressure after 2 hours was 164/93. The symptoms resolved after 30 minutes. Troponins were negative, and EKG did not show any ST segment, T, and Q wave changes.
Third time (day 13 since admission)	Around 10:30 pm, blood pressure was 242/119 with headache, left-sided upper extremity weakness with tingling, and chest pain.	Already took losartan 100 mg in the evening. Transdermal nitroglycerin patch 0.2 mg/h was given. Elevate the head of the bed to 30 degrees. Troponins and EKG were ordered.	Recheck blood pressure after 1 hour was 171/97. Recheck blood pressure after 4 hours was 148/80. The symptoms resolved after 1 hour. Troponins were negative, and EKG did not show any ST segment, T, and Q wave changes.
Fourth time (day 14 since admission)	Awoken at 1 am. Blood pressure was 236/106 with headache, left-sided upper extremity weakness and tingling.	Already took losartan 100 mg and transdermal nitroglycerin patch 0.2 mg/h. Nitro patch removed and nitro paste was given for more rapid lowering of blood pressure.	Recheck blood pressure after 1 hour was 163/103. The symptoms resolved after 30 minutes of application of nitro paste.
Fifth time (day 16 since admission)	Bedtime blood pressure was 233/109 with left-sided chest pain.	Already took losartan 100 mg and diltiazem 30 mg in the evening. Transdermal nitroglycerin patch 0.2 mg/h was given. Troponins and EKG were ordered.	Recheck blood pressure after 1 hour was 146/76. Recheck blood pressure after 2 hours was 164/92. Chest pain resolved 15 minutes after the nitro patch was given. Troponins were negative, and EKG did not show any ST segment, T, and Q wave changes.

Work-ups for other potential causes of autonomic failure conducted on day 16 included the following results: serum tests were negative for Lyme disease antibodies, human immunodeficiency virus (HIV) antibodies, rapid plasma reagin, and heavy metals such as arsenic, lead, and thallium. Vitamin B12 levels were 710 picograms per milliliter (pg/mL) (normal range: >300 pg/mL). Serum protein electrophoresis showed normal total protein and immunoglobulins, with a negative M-spike. Serum free light chains revealed a mildly elevated kappa-lambda free light chain ratio of 1.83 (normal range: 0.26-1.65). The DYS2 Mayo Clinic panel for idiopathic dysautonomia was sent but was reported as contaminated. The patient was scheduled for follow-up with the outpatient autonomic clinic after discharge for further work-up.

**Figure 1:**
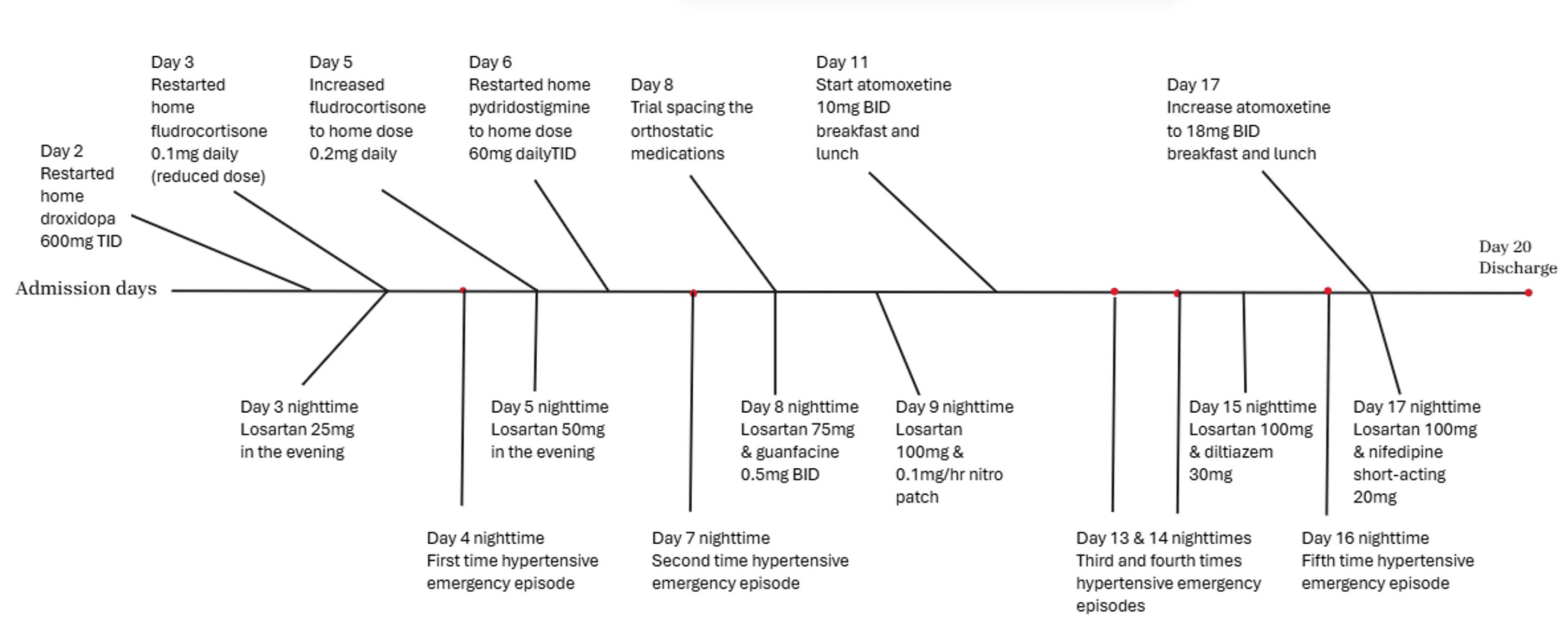
Timeline of the inpatient management of the orthostatic hypotension and the nocturnal supine hypertension mg: milligrams; mg/h: milligrams per hour; TID: three times daily; BID: two times daily.

After 19 days of admission, the patient's symptoms of orthostatic hypotension had significantly improved for at least 48 hours, and supine blood pressure control was adequate for two consecutive nights. The patient was discharged home with conservative measures, including the use of an abdominal binder and compression stockings (to be removed only at bedtime). The medication regimen upon discharge included droxidopa 600 mg three times daily at 6 am, 12 pm, and 6 pm; fludrocortisone 0.2 mg once daily at 6 am; pyridostigmine 60 mg three times daily at 7 am, 12 pm, and 5 pm; and atomoxetine 18 mg twice daily at breakfast and lunch. To manage supine hypertension, the patient was advised to elevate the head of the bed at least 30 degrees at all times and to avoid vasopressors 3 hours before bedtime. Additionally, losartan 100 mg and short-acting nifedipine 20 mg were prescribed at bedtime. Follow-up appointments with the autonomic team and endocrinology (for improved glycemic control) were scheduled. Figure 1 provides a complete timeline of the inpatient management of orthostatic hypotension and supine hypertension.

## Discussion

Diabetic CAN is observed in approximately one-fourth of patients with type 1 diabetes and one-third of those with type 2 diabetes. It is associated with an increased risk of coronary artery disease and stroke. The prevalence of CAN increases with age, duration of diabetes, and poor glycemic control. In CAN, diabetic complications damage the autonomic nerve fibers that innervate the heart and blood vessels, leading to abnormalities in heart rate control and vascular dynamics. Clinical manifestations of CAN include orthostatic hypotension, loss of heart rate variability, silent myocardial infarction due to cardiac denervation, orthostatic tachycardia, and bradycardia syndromes [3]. Multiple studies have demonstrated an increased mortality rate among diabetics with CAN. While cardiovascular disease associated with diabetes may overlap with CAN in some patients, evidence suggests that CAN is an independent factor contributing to poor prognosis. The overall mortality rate over a 10-year period was approximately 29% in diabetics with CAN, compared to 6% in those without evidence of CAN [4].

The CAN Subcommittee of the Toronto Consensus Panel recommends universal screening for CAN in all patients with diabetes, utilizing four gold standard tests (CARTs): heart rate response to deep breathing, standing, Valsalva maneuver, and blood pressure response to standing. Based on the abnormalities identified in these tests (CARTs), CAN can be categorized into three levels: 1. possible or early CAN (1 abnormal CART), 2. definite or confirmed CAN (2-3 abnormal CARTs), and 3. severe advanced CAN (orthostatic hypotension along with 2 or more abnormal CARTs). As for our patient, he has two abnormal CARTs (abnormal heart rate and blood pressure response to standing) along with symptomatic orthostatic hypotension, which fits into the category of severe advanced CAN. Early interventions such as dietary and exercise modifications, along with glycemic control optimization, are crucial following diagnosis, as these interventions have been associated with improvements in cardiac autonomic function. Management should not be postponed until symptoms and signs of CAN manifest, as these often indicate advanced stages of the condition, at which point reversibility may be lacking [5].

Orthostatic hypotension is defined as a sustained reduction of at least 20 mmHg in SBP or at least 10 mmHg in DBP within 3 minutes of standing or tilting the head up to at least 60 degrees on a tilt table. In patients with supine hypertension, a sustained reduction of at least 30 mmHg in SBP is more appropriate, as the magnitude of the blood pressure drop depends on the baseline blood pressure [6]. The prevalence of orthostatic hypotension is age-dependent, ranging from 5-11% in middle-aged individuals to 30% or higher in the elderly. The highest prevalence is observed in patients with hypertension (15% to 30%), diabetes (15% to 25%), or Parkinson’s disease (approximately 50%) [7]. In a survey conducted at a tertiary referral center, 38% of patients with orthostasis exhibited no evidence of generalized autonomic dysfunction, 35% had diabetic neuropathy or paraneoplastic syndromes as probable causes of autonomic failure, and 27% had primary autonomic failure [8].

nOH resulting from severe advanced diabetic CAN is attributed to baroreflex dysfunction, as postganglionic sympathetic neurons fail to release norepinephrine appropriately. In these individuals, blood pressure drops upon standing due to inadequate compensatory vasoconstriction to counteract the gravitational pull of blood to the lower limbs. This insufficient vasoconstriction can lead to symptoms of organ hypoperfusion, including lightheadedness, dizziness, visual disturbances, syncope, subtle cognitive slowing, and fatigue during standing or exertion [9].

Management of nOH is a stepwise process aimed at reducing symptom burden and minimizing the risk of falls. Initial strategies include nonpharmacological measures and possible discontinuation of medications that may exacerbate symptoms. Nonpharmacological interventions include 1. Increased salt intake (adding 1-2 teaspoons, or 2.3-4.6 g of salt per day to the normal diet); 2. increased water intake (up to 2-3 liters per day); 3. elevating the head of the bed 6-9 inches to avoid nocturnal supine hypertension, which can precipitate nocturnal diuresis; 4. engaging in physical conditioning, such as lower body strength training and moderate (non-strenuous) activity; 5. avoiding increased core body temperature from strenuous exercise and hot tubs, which can cause peripheral vasodilation; 6. using an abdominal binder or waist-high compression stockings that exert 30-40 mmHg of pressure to reduce venous pooling; 7. consuming smaller, more frequent meals to prevent postprandial hypotension associated with larger meals; and 8. correcting anemia and vitamin B12 deficiencies to improve symptoms and postural instability, respectively [10]. For a review of medications that may worsen orthostatic symptoms, see Table 5.

**Table 5:** Common medication classes and examples of medications that can exacerbate the orthostatic hypotension

Classes of medications	Common examples
Angiotensin-converting enzyme inhibitors	Lisinopril, enalapril, ramipril
Angiotensin receptor blockers	Losartan, valsartan
Angiotensin receptor-neprilysin inhibitor	Sacubitril-valsartan
Beta-blockers	Metoprolol, carvedilol, propranolol
Calcium channel blockers	Amlodipine, nifedipine, diltiazem, verpamil
Diuretics	Furosemide, thiazides, spironolactone
Nitrates	Isosorbide dinitrate, nitroglycerin
Alpha agonists	Tamsulosin, prazosin, clonidine
Vasodilators	Hydralazine
Phosphodiesterase inhibitors	Sildenafil, tadalafil
Dopaminergic agents	Levodopa, ropinirole
Antidepressants	Imipramine, amitriptyline, nortriptyline
Anticholinergics	Atropine, hyoscyamine

If there is no adequate improvement in nOH with nonpharmacological means, or if patients present with syncope or symptomatic falls, pharmacological measures may be appropriate in conjunction with the nonpharmacological strategies mentioned above. Current pharmacological management is based on two primary approaches: 1. expanding intravascular volume and 2. increasing peripheral vascular resistance through sympathetic enhancement. Physicians can choose either approach or a combination, depending on the specifics of the patient’s condition.

For patients with a known partial response to increased salt and water intake, initial therapy can begin with low-dose fludrocortisone to promote intravascular volume expansion. For patients with no known response to increased salt and water intake, measuring plasma norepinephrine levels can help guide the sympathetic enhancement strategy. A plasma norepinephrine level lower than 220 picograms per milliliter (pg/mL), indicative of severe widespread postganglionic sympathetic denervation, may predict a better response to medications like droxidopa or midodrine. Conversely, normal or high norepinephrine levels greater than 220 pg/mL, suggesting less pronounced sympathetic denervation, may indicate a better response to norepinephrine reuptake inhibitors such as atomoxetine. In patients with refractory nOH, cholinesterase inhibitors, such as pyridostigmine, may be added as a complementary therapy to the aforementioned medications. Alternatively, norepinephrine reuptake inhibitors (e.g., atomoxetine) could potentially be combined with droxidopa or midodrine, with or without the addition of fludrocortisone or pyridostigmine. However, data on the safety of the combined use of most of these medications, both in the short-term and long-term, is limited [11]. Table 6 outlines the mainstream pharmacological management options for nOH, including their mechanisms of action and potential side effects.

**Table 6:** Pharmacological management for nOH with mechanisms of action and side-effects of mainstream medications nOH: neurogenic orthostatic hypotension; mg/day: milligrams per day; mg: milligrams; BID: two times a day; TID: three times a day.

Pharmacological management and recommended dosage	Mechanism of action	Side effects
Fludrocortisone; 0.05 mg/day-0.2 mg/day (no benefit with higher dosages but can result in fluid overload and congestive heart failure)	Intravascular volume expansion via salt and water retention, synthetic mineralocorticoid, requires at least 7 days to see the effect	Supine hypertension, hypokalemia (advise patients with potassium-rich diet and monitor serum potassium 3-4 monthly), acceleration of kidney disease [12]
Midodrine [13]; 2.5-15 mg BID or TID (last dose to be 3-4 hours before bedtime)	Sympathetic enhancement as a direct alpha-1 adrenergic agonist; causes vasoconstriction to increase peripheral vascular resistance, and raises blood pressure without increasing the heart rate	Supine hypertension, bradycardia
Droxidopa [14]; 100-600 mg TID (last dose to be 3-4 hours before bedtime)	Sympathetic enhancement by increased conversion of norepinephrine by dopa‑decarboxylase, more pressor response in low plasma norepinephrine levels	Supine hypertension, headache, and nausea
Atomoxetine [15]; 10-18 mg BID	Sympathetic enhancement by prolonging bioavailability of norepinephrine at the neurovascular junction as norepinephrine reuptake inhibitor; more pressor response in patients with high plasma norepinephrine levels	Supine hypertension, tachycardia, insomnia, and headache
Pyridostigmine [16]; 30-60 mg BID or TID	Indirect sympathetic enhancement by reversible acetylcholinesterase inhibition; enhances ganglionic cholinergic neurotransmission, stimulating the release of norepinephrine; small pressor effect	Bradycardia, nausea, and increased peristalsis

In this case, the patient required strict adherence to both nonpharmacological and pharmacological measures, which involved a regimen of four medications: droxidopa, fludrocortisone, pyridostigmine, and atomoxetine. It is noteworthy that the patient had already been on three of these medications prior to hospitalization. Furthermore, the orthostatic vital signs and symptoms improved during the inpatient stay, particularly once nocturnal supine hypertension was effectively addressed before discharge.

Approximately 50% of patients with nOH develop neurogenic supine hypertension (nSH), defined as a sustained elevation of SBP of at least 140 mmHg and/or DBP of at least 90 mmHg in the supine position. This condition is presumed to result from central or peripheral autonomic lesions [17]. nSH occurs due to the loss of the physiological nocturnal blood pressure drop (a decrease of ≥10% from the mean daytime blood pressure) during the night. Though often asymptomatic, some patients with nSH may report non-specific symptoms such as headaches. It can be categorized into 1. mild if SBP is 140-159 mmHg or DBP is 90-99 mmHg; 2. moderate if SBP is 160-179 mmHg or DBP is 100-109 mmHg; and 3. severe if symptomatic or if SBP is more than or equal to 180 mmHg or DBP is more than or equal to 110 mmHg. The primary concern with nSH is the exacerbation of pressure natriuresis during sleep, leading to nocturia and volume depletion at night, which can worsen orthostatic hypotension upon waking [18].

The management of nocturnal supine hypertension in patients with orthostatic hypotension is indeed challenging and requires a step-wise approach. The first step involves nonpharmacological means such as 1. elevating the head of bed at least 6 to 9 inches to reduce blood pressure and nocturnal natriuresis; 2. avoiding medications for orthostatic hypotension and water intake boluses close to bedtime; 3. refrain from abdominal binders or compression garments when supine; and 4. encouraging a light snack, preferably low in salt before bedtime for mild postprandial hypotension effect [19].

If the patient remains in severe nocturnal supine hypertension or is symptomatic despite nonpharmacological measures, pharmacological management may be necessary. Nocturnal supine hypertension can be effectively controlled with several short-acting antihypertensives administered at bedtime. Options include nitroglycerin patches, immediate-release nifedipine, losartan, sildenafil, clonidine, and nebivolol. Patients should be cautioned about the potential risk of falls and be encouraged to implement fall prevention strategies when getting up at night to urinate, especially if they are receiving pharmacological treatment for supine hypertension. In refractory cases, guanfacine or clonidine patches may be beneficial, particularly if baroreflex dysfunction is the primary cause [20]. Regular follow-up and monitoring are essential, as these treatments can potentially worsen orthostatic hypotension and increase the risk of falls. Table 7 provides a comprehensive overview of various pharmacological options for managing nocturnal supine hypertension.

**Table 7:** Nocturnal supine hypertension: pharmacological treatments, their mechanisms of actions, and side-effects mg/h: milligrams per hour; mg: milligrams.

Pharmacological management and recommended dosage	Mechanism of action	Side-effects
Nitroglycerin patch; 0.1 mg/h at bedtime and removed in the morning	Vasodilator effect on peripheral veins and arteries via nitric oxide	Can worsen orthostatic hypotension along with an increase in nocturnal natriuresis, and headache
Nifedipine immediate-release 30 mg at bedtime	Reduce arterial blood pressure by reducing peripheral vascular resistance	Can worsen orthostatic hypotension along with an increase in nocturnal natriuresis, peripheral edema, and headache
Losartan 50 mg at bedtime	Blocks the vasoconstrictor and aldosterone-secreting effects of angiotensin by blocking angiotensin receptor	Can worsen orthostatic hypotension, contraindicated in patients with congestive heart failure
Sildenafil 25 mg at bedtime	Vasodilatation in the systemic circulation by relaxing smooth muscle in the vasculature	Can worsen orthostatic hypotension along with an increase in nocturnal natriuresis, headaches, vision color changes, and flushing
Clonidine 0.1 mg at bedtime	Reduce sympathetic outflow by stimulating alpha-2 adrenoreceptors which activate an inhibitory neuron	Can worsen orthostatic hypotension, bradycardia, drowsiness, and headache
Nebivolol 5 mg at bedtime	Highly selective beta-1 adrenergic receptor blocker and produces nitric oxide-dependent vasodilation which would reduce systemic vascular resistance	Can worsen orthostatic hypotension along with an increase in nocturnal natriuresis, and bradycardia

A hypertensive emergency is defined as a situation where a patient presents with severely elevated blood pressure (usually SBP greater than 180 mmHg and DBP greater than 120 mmHg) accompanied by symptoms of target organ damage. In this case, the patient’s nocturnal supine hypertension was severe and symptomatic, leading to episodes that mimicked hypertensive emergencies, with focal neurological deficits. The patient's initial presentation with left hemiparesis, hemihypoesthesia, left central facial palsy, and dysarthria, for which he received tenecteplase, was assessed to be related to the hypertensive emergency caused by the severe nocturnal supine hypertension as acute stroke was ruled out as there were no ischemic changes on brain MRI. During the hypertensive emergency episodes experienced while inpatient, the symptoms - headaches, left arm weakness with tingling, and chest pain - were consistent across multiple episodes and resolved upon blood pressure reduction. 

There is currently no definitive evidence to guide the selection of specific medications for managing supine hypertension in patients with nOH. The choice of medication typically depends on the patient’s underlying conditions. For example, in patients with diabetes, chronic kidney disease, or proteinuria, losartan may be a better choice, while nebivolol is preferred in those with coronary artery disease. Tolerance to medications also plays a critical role in the decision-making process. The ideal treatment for nocturnal supine hypertension associated with orthostatic hypotension in CAN should aim to control nocturnal supine hypertension, reduce natriuresis, and improve daytime orthostatic symptoms. Among the medications listed in the previous table, only clonidine and losartan effectively reduced nighttime diuresis; however, neither improved morning orthostatic tolerance and both have the potential to worsen associated orthostatic hypotension. Therefore, there are no medications that fulfill all criteria for the ideal treatment of nocturnal supine hypertension in patients with nOH [21].

Given the complexity of managing both orthostatic and nocturnal hypertension, regular and frequent follow-ups with the autonomic team are essential. This follow-up is necessary to monitor symptoms, assess the effectiveness of medications, and identify any potential side effects.

## Conclusions

In patients with severe advanced diabetic CAN, management of nOH follows a stepwise approach, starting with nonpharmacological interventions such as increased salt and water intake and using abdominal binders or compression stockings. Pharmacological measures with single or combined therapies may be employed if necessary. The choice of pharmacological management depends on the patient's response to increased salt and water intake and plasma norepinephrine levels although not routinely measured in clinical practice. Although the combined use of medications may be beneficial for refractory symptoms, data regarding the safety of such combinations for orthostatic hypotension for both short-term and long-term use remains limited. Additionally, the management of orthostatic hypotension can exacerbate coexisting nocturnal supine hypertension, especially with combined medications.

Management of nocturnal supine hypertension should also be stepwise, initiating with nonpharmacological strategies such as elevation of the head of the bed and avoiding orthostatic medications close to bedtime, followed by pharmacological intervention if necessary. Currently, there is no definitive evidence for definitive management of supine hypertension in patients with severe orthostatic hypotension. Further research into novel neuromodulatory pharmacological or interventional therapies that would satisfy the requirements for the ideal treatment of orthostatic hypotension or nocturnal supine hypertension in this population is warranted. It is also important to emphasize the need for interdisciplinary research involving cardiology, neurology, and pharmacology in developing those novel therapies. 
